# The genome sequence of a conopid fly, *Thecophora atra *(Fabricius, 1775)

**DOI:** 10.12688/wellcomeopenres.19902.1

**Published:** 2023-08-21

**Authors:** Ryan Mitchell, Steven Falk, Sam Thomas, Olga Sivell, Duncan Sivell, Natural History Museum Genome Acquisition Lab, University of Oxford and Wytham Woods Genome Acquisition Lab, Darwin Tree of Life Barcoding collective, Wellcome Sanger Institute Tree of Life programme, Wellcome Sanger Institute Scientific Operations: DNA Pipelines collective, Tree of Life Core Informatics collective, Darwin Tree of Life Consortium

**Affiliations:** 1 Independent researcher, Sliigo, Ireland; 2 Independent researcher, Kenilworth, England, UK; 3 Natural History Museum, London, England, UK

**Keywords:** Thecophora atra, conopid fly, genome sequence, chromosomal, Diptera

## Abstract

We present a genome assembly from an individual male *Thecophora atra* (a Conopid fly; Arthropoda; Insecta; Diptera; Conopidae). The genome sequence is 354.2 megabases in span. Most of the assembly is scaffolded into 5 chromosomal pseudomolecules, including the X and Y sex chromosomes. The mitochondrial genome has also been assembled and is 17.3 kilobases in length. Gene annotation of this assembly on Ensembl identified 30,620 protein coding genes.

## Species taxonomy

Eukaryota; Metazoa; Eumetazoa; Bilateria; Protostomia; Ecdysozoa; Panarthropoda; Arthropoda; Mandibulata; Pancrustacea; Hexapoda; Insecta; Dicondylia; Pterygota; Neoptera; Endopterygota; Diptera; Brachycera; Muscomorpha; Eremoneura; Cyclorrhapha; Schizophora; Acalyptratae; Conopoidea; Conopidae; Myopinae; *Thecophor*a; *Thecophora atra* (Fabricius, 1775) (NCBI:txid1219171).

## Background

*Thecophora atra* (Fabricius, 1775) is a medium sized black fly from the family Conopidae, occasionally called thick-headed flies. It is one of three species from genus *Thecophora* Rondani, 1845 occurring in Britain ( Ransom *et al.*, 2015; Whitehead, 2022). It differs from *Thecophora fulvipes* (Robineau-Desvoidy, 1830) by being slightly smaller in size ( *T. atra* is 4–7 mm, in contrast to 5–9 mm of *T. fulvipes*) and having scarcer dusting on the abdomen. The legs of *T. atra* are usually black with yellow “knees” (distal tip of the femora and proximal tip of the tibia) and a yellow basal half of the hind femora, while in *T. fulvipes* all the femora are entirely yellow or orange-brown (the hind one at least in basal two-thirds). The leg colouration is somewhat variable and some *T. atra* may have some yellow at the base or apex of front and/or mid femora ( Ransom *et al.*, 2015; Smith, 1969).

*Thecophora cinerascens* (Meigen, 1804) was discovered in the Channel Islands in 2015 and in Wales in 2019, adding another species to the British list. Although similar in size and appearance to *T. atra,* the females of both species can be reliably separated by the shape of theca ( Ransom *et al.*, 2015; Whitehead, 2022). Male *T. atra* and *T. cinerascens* are very difficult to separate. The specimen used for sequencing was confirmed as *T. atra* using DNA barcodes.

*Thecophora atra* is oviparous and its larvae are internal parasites of halictid bees. Usually, a single egg is laid in flight into a host’s abdomen. The white and smooth larva feeds on the insides of the abdomen during which time the host is active. The larva then becomes tapered anteriorly (in the third instar) and feeds on the contents of the thorax through the petiole resulting in the death of the host. Pupation occurs soon after, inside the host’s abdomen ( Smith, 1966).

The hosts recorded outside Britain include *Halictus* and *Lasioglossum* species. In Britain, *T. atra* has been observed around colonies of *Lasioglossum morio* and *Halictus* spp., but parasitism of these species has not been confirmed through rearing ( Baldock & Early, 2015; Smith, 1969).

The adults can be seen on flowers, particularly on ragwort *Jacobaea* spp. (= *Senecio* spp.), rough hawkbit *Leontodon hispidus*, devil’s-bit scabious *Succisa pratensis* (= *Scabiosa succisa*), water mint *Mentha aquatica*, hawkweed *Hieracium* spp., speedwells *Veronica* spp., common rock-rose *Helianthemum nummularium* and thistles ( Baldock & Early, 2015; Smith, 1969). *Thecophora atra* occurs on chalk, calcareous or basi-neutral habitats such as species-rich grasslands, and can also be found on or near the coast ( Baldock & Early, 2015; Ransom *et al.*, 2015). *Thecophora atra* is widely distributed in Britain and Ireland, more common in southern Britain, scarcer in northern England, with few records from Scotland ( Ransom *et al.*, 2015; Smith, 1969). *T. atra* is on the wing from May to October, peaking in August ( Baldock & Early, 2015; Smith, 1969).

The high-quality genome of a conopid fly *Thecophora atra* was sequenced based on one male specimen from Hartslock Nature Reserve. It will aid research on the taxonomy, phylogeny and biology of this and related taxa. The genome of *T. atra* was sequenced as part of the Darwin Tree of Life Project, a collaborative effort to sequence all named eukaryotic species in the Atlantic Archipelago of Britain and Ireland.

## Genome sequence report

**Figure 1.**
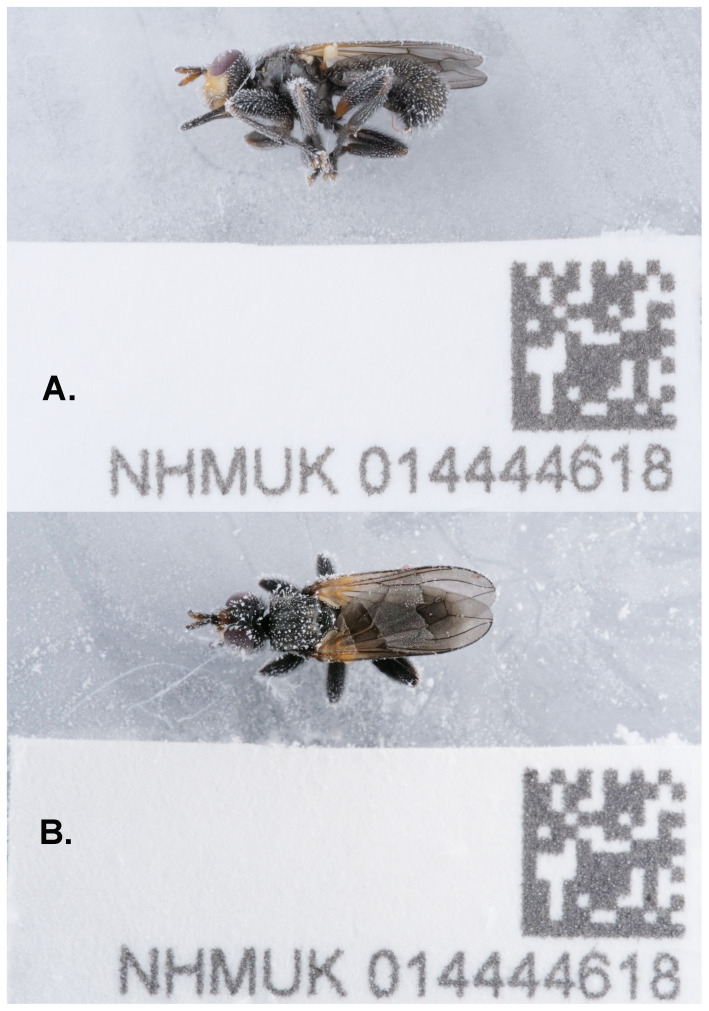
Photographs of the *Thecophora atra* (specimen ID NHMUK014444618, ToLID idTheAtra2) specimen used for genome sequencing. **A**. The specimen in lateral view. **B**. The specimen in dorsal view. Photographs by Olga Sivell.

The genome was sequenced from one male *Thecophora atra* ( Figure 1) collected from Hartslock Reserve, Oxfordshire (51.51, –1.11). A total of 44-fold coverage in Pacific Biosciences single-molecule HiFi long reads was generated. Primary assembly contigs were scaffolded with chromosome conformation Hi-C data. Manual assembly curation corrected 46 missing joins or misjoins and removed two haplotypic duplications, reducing the assembly length by 0.66% and the scaffold number by 52.17%, and increasing the scaffold N50 by 122%.

**Figure 2.**
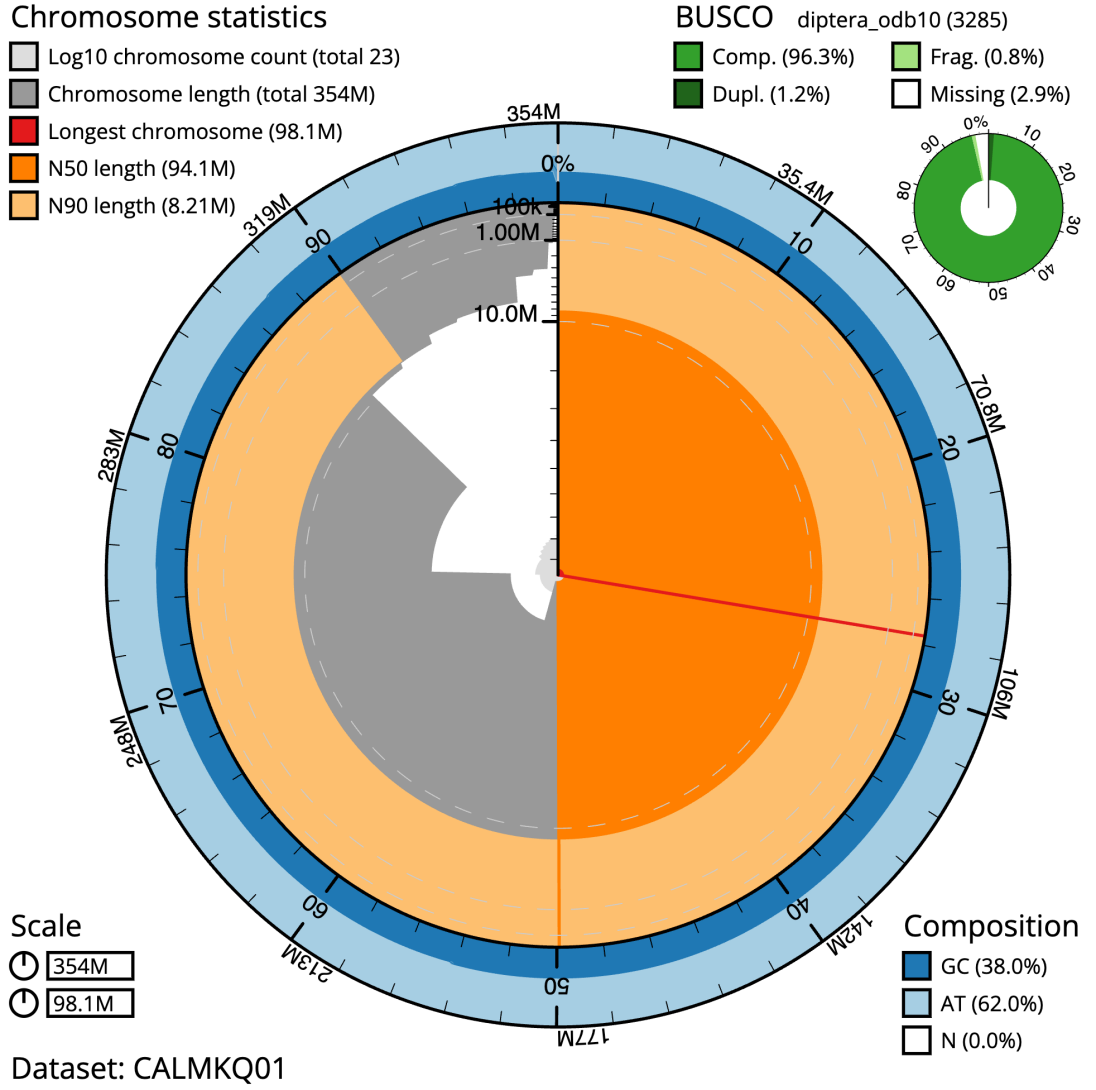
Genome assembly of *Thecophora atra*, idTheAtra2.1: metrics. The BlobToolKit Snailplot shows N50 metrics and BUSCO gene completeness. The main plot is divided into 1,000 size-ordered bins around the circumference with each bin representing 0.1% of the 354,246,487 bp assembly. The distribution of scaffold lengths is shown in dark grey with the plot radius scaled to the longest scaffold present in the assembly (98,076,447 bp, shown in red). Orange and pale-orange arcs show the N50 and N90 scaffold lengths (94,083,503 and 8,213,565 bp), respectively. The pale grey spiral shows the cumulative scaffold count on a log scale with white scale lines showing successive orders of magnitude. The blue and pale-blue area around the outside of the plot shows the distribution of GC, AT and N percentages in the same bins as the inner plot. A summary of complete, fragmented, duplicated and missing BUSCO genes in the diptera_odb10 set is shown in the top right. An interactive version of this figure is available at https://blobtoolkit.genomehubs.org/view/Thecophora%20atra/dataset/CALMKQ01/snail.

The final assembly has a total length of 354.2 Mb in 22 sequence scaffolds with a scaffold N50 of 94.1 Mb ( Table 1). Most (99.94%) of the assembly sequence was assigned to 5 chromosomal-level scaffolds, representing three autosomes and the X and Y sex chromosomes. The Y chromosome was identified based on read coverage against the Hi-C reads from a different specimen. Chromosome-scale scaffolds confirmed by the Hi-C data are named in order of size ( Figure 2– Figure 5; Table 2). While not fully phased, the assembly deposited is of one haplotype. Contigs corresponding to the second haplotype have also been deposited. The mitochondrial genome was also assembled and can be found as a contig within the multifasta file of the genome submission.

**Table 1.** Genome data for *Thecophora atra*, idTheAtra2.1.

Project accession data		
Assembly identifier	idTheAtra2.1	
Species	*Thecophora atra*	
Specimen	idTheAtra2	
NCBI taxonomy ID	1219171	
BioProject	PRJEB51036	
BioSample ID	SAMEA7849382	
Isolate information	idTheAtra2: head (DNA sequencing) idTheAtra3: thorax (Hi-C data) idTheAtra1: whole organism (RNA sequencing)	
Assembly metrics *		*Benchmark*
Consensus quality (QV)	65	*≥ 50*
*k*-mer completeness	100%	*≥ 95%*
BUSCO **	C:96.3%[S:95.2%,D:1.2%], F:0.8%,M:2.9%,n:3,285	*C ≥ 95%*
Percentage of assembly mapped to chromosomes	99.94%	*≥ 95%*
Sex chromosomes	X and Y chromosomes	*localised homologous pairs*
Organelles	Mitochondrial genome assembled	*complete single alleles*
Raw data accessions		
PacificBiosciences SEQUEL II	ERR8978460	
Hi-C Illumina	ERR8702824	
PolyA RNA-Seq Illumina	ERR10123684	
Genome assembly		
Assembly accession	GCA_937620795.1	
*Accession of alternate haplotype*	GCA_937641085.1	
Span (Mb)	354.2	
Number of contigs	94	
Contig N50 length (Mb)	7.7	
Number of scaffolds	22	
Scaffold N50 length (Mb)	94.1	
Longest scaffold (Mb)	98.1	
Genome annotation		
Number of protein-coding genes	30,620	
Number of gene transcripts	31,353	

* Assembly metric benchmarks are adapted from column VGP-2020 of “Table 1: Proposed standards and metrics for defining genome assembly quality” from ( Rhie *et al.*, 2021). ** BUSCO scores based on the diptera_odb10 BUSCO set using v5.3.2. C = complete [S = single copy, D = duplicated], F = fragmented, M = missing, n = number of orthologues in comparison. A full set of BUSCO scores is available at https://blobtoolkit.genomehubs.org/view/Thecophora%20atra/dataset/CALMKQ01/busco.

**Figure 3.**
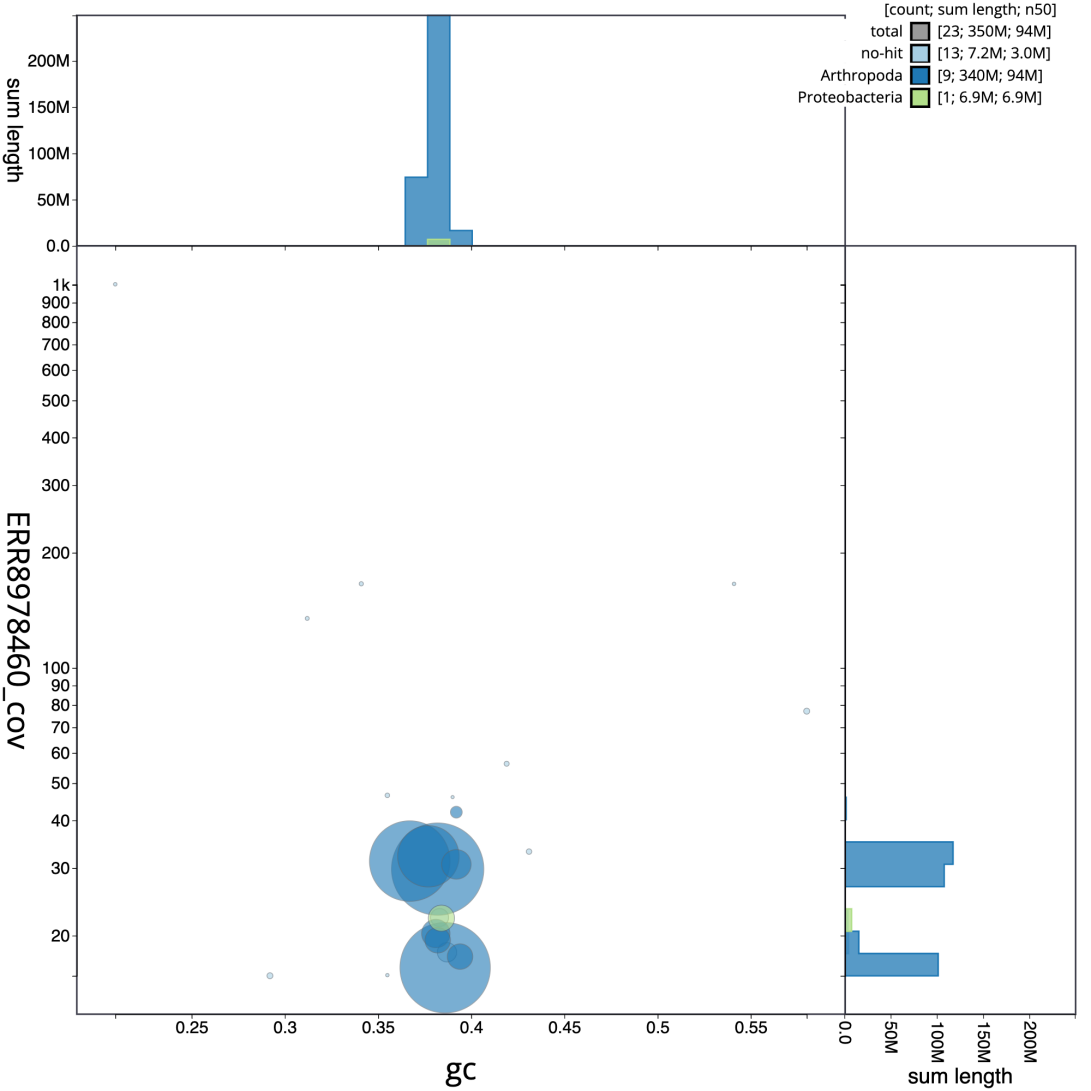
Genome assembly of *Thecophora atra*, idTheAtra2.1: BlobToolKit GC-coverage plot. Scaffolds are coloured by phylum. Circles are sized in proportion to scaffold length. Histograms show the distribution of scaffold length sum along each axis. An interactive version of this figure is available at https://blobtoolkit.genomehubs.org/view/Thecophora%20atra/dataset/CALMKQ01/blob.

**Figure 4.**
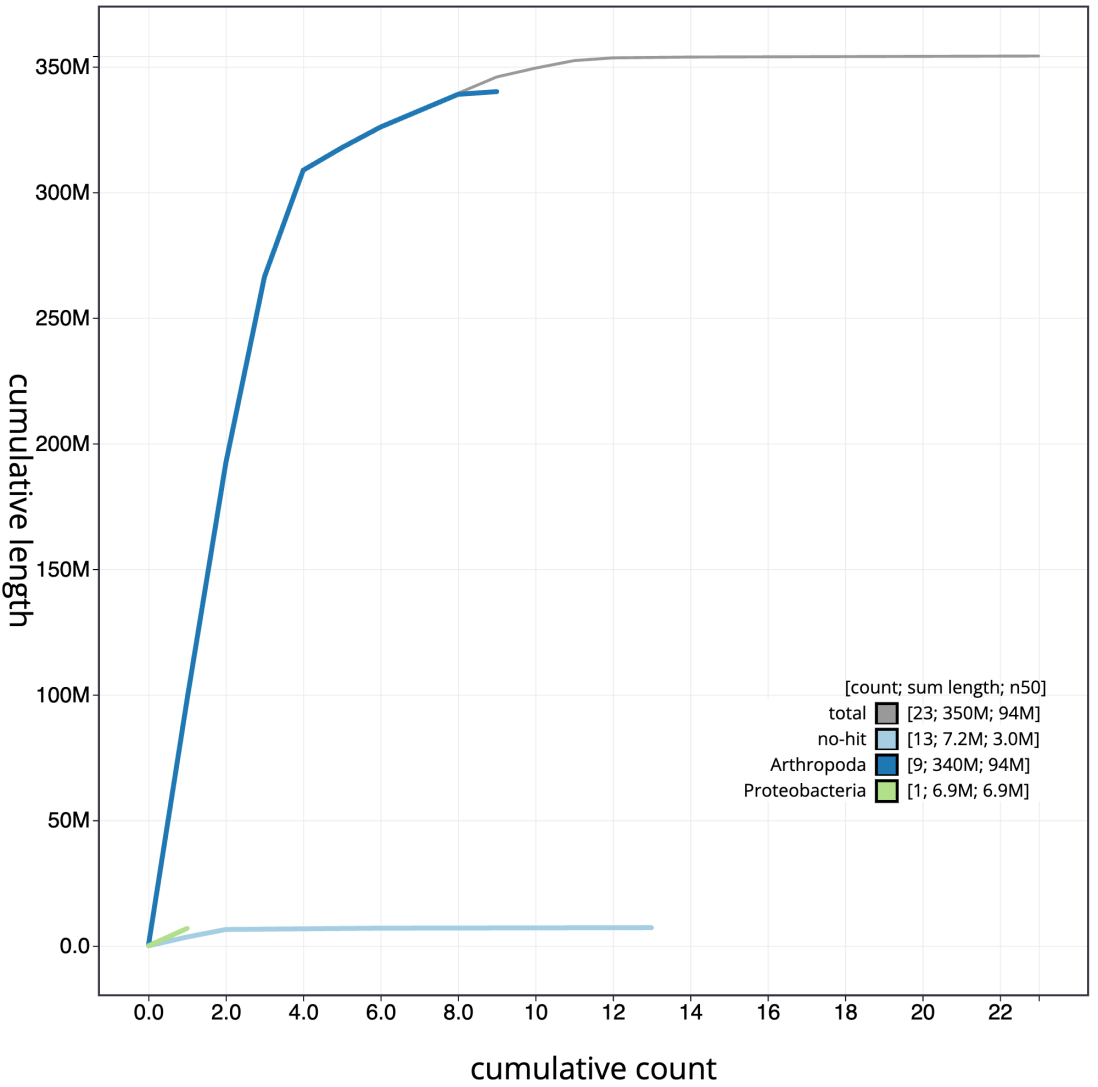
Genome assembly of *Thecophora atra*, idTheAtra2.1: BlobToolKit cumulative sequence plot. The grey line shows cumulative length for all scaffolds. Coloured lines show cumulative lengths of scaffolds assigned to each phylum using the buscogenes taxrule. An interactive version of this figure is available at https://blobtoolkit.genomehubs.org/view/Thecophora%20atra/dataset/CALMKQ01/cumulative.

**Figure 5.**
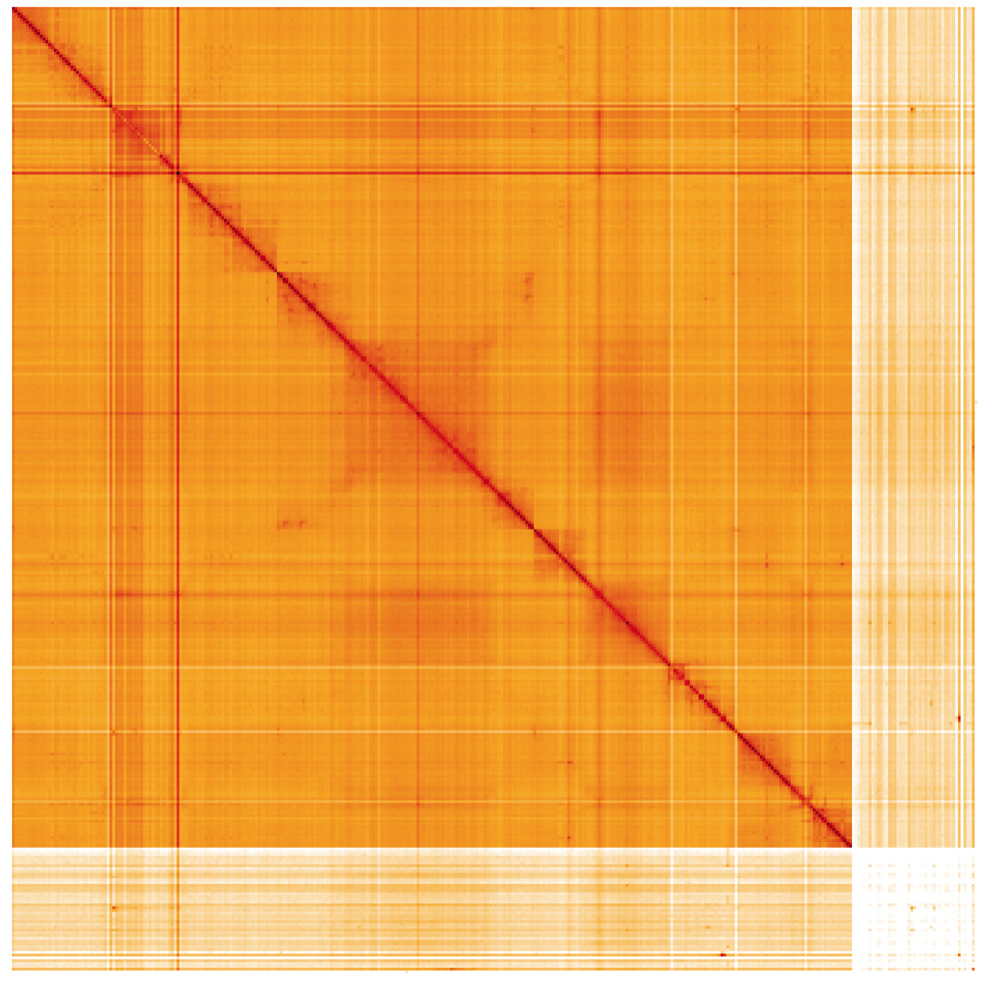
Genome assembly of *Thecophora atra*, idTheAtra2.1: Hi-C contact map of the idTheAtra2.1 assembly, visualised using HiGlass. Chromosomes are shown in order of size from left to right and top to bottom. An interactive version of this figure may be viewed at https://genome-note-higlass.tol.sanger.ac.uk/l/?d=LDkQ8LYLT9KdKtYbH37mvw.

**Table 2.** Chromosomal pseudomolecules in the genome assembly of *Thecophora atra*, idTheAtra2.

INSDC accession	Chromosome	Length (Mb)	GC%
OW569397.1	1	98.08	38.0
OW569399.1	2	74.24	36.5
OW569401.1	3	42.38	37.5
OW569398.1	X	94.08	38.5
OW569400.1	Y	8.99	39.0
OW569402.1	MT	0.02	21.0

The estimated Quality Value (QV) of the final assembly is 65 with *k*-mer completeness of 100%, and the assembly has a BUSCO v5.3.2 completeness of 96.3% (single = 95.2%, duplicated = 1.2%), using the diptera_odb10 reference set ( *n* = 3,285).

Metadata for specimens, spectral estimates, sequencing runs, contaminants and pre-curation assembly statistics can be found at https://links.tol.sanger.ac.uk/species/1219171.

## Genome annotation report

The *Thecophora atra* genome assembly (GCA_937620795.1) was annotated using the Ensembl rapid annotation pipeline ( Table 1; https://rapid.ensembl.org/Thecophora_atra_GCA_937620795.1/Info/Index). The resulting annotation includes 31,353 transcribed mRNAs from 30,620 protein-coding genes.

## Methods

### Sample acquisition and nucleic acid extraction

*Thecophora atra* specimens (idTheAtra2 and idTheAtra3 NHMUK014444821) were collected from Hartslock Nature Reserve, Oxfordshire, UK (latitude 51.51, longitude –1.11) on 2020-08-20 using an aerial net. The specimens were collected and identified by Ryan Mitchell (Natural History Museum) and preserved on dry ice.

The specimen used for RNA sequencing (specimen ID Ox000734, ToLID idTheAtra1) was collected from Wytham Woods, Oxfordshire (biological vice-county Berkshire), UK (51.766, –1.309) on 2020-08-03 by netting. This specimen was collected and identified by Steven Falk (independent researcher).

DNA was extracted at the Tree of Life laboratory, Wellcome Sanger Institute (WSI). The idTheAtra2 sample was weighed and dissected on dry. Head tissue was disrupted using a Nippi Powermasher fitted with a BioMasher pestle. High molecular weight (HMW) DNA was extracted using the Qiagen MagAttract HMW DNA extraction kit. HMW DNA was sheared into an average fragment size of 12–20 kb in a Megaruptor 3 system with speed setting 30. Sheared DNA was purified by solid-phase reversible immobilisation using AMPure PB beads with a 1.8X ratio of beads to sample to remove the shorter fragments and concentrate the DNA sample. The concentration of the sheared and purified DNA was assessed using a Nanodrop spectrophotometer and Qubit Fluorometer and Qubit dsDNA High Sensitivity Assay kit. Fragment size distribution was evaluated by running the sample on the FemtoPulse system.

RNA was extracted from whole organism tissue of idTheAtra1 in the Tree of Life Laboratory at the WSI using TRIzol, according to the manufacturer’s instructions. RNA was then eluted in 50 μl RNAse-free water and its concentration assessed using a Nanodrop spectrophotometer and Qubit Fluorometer using the Qubit RNA Broad-Range (BR) Assay kit. Analysis of the integrity of the RNA was done using Agilent RNA 6000 Pico Kit and Eukaryotic Total RNA assay.

### Sequencing

Pacific Biosciences HiFi circular consensus DNA sequencing libraries were constructed according to the manufacturers’ instructions. Poly(A) RNA-Seq libraries were constructed using the NEB Ultra II RNA Library Prep kit. DNA and RNA sequencing was performed by the Scientific Operations core at the WSI on Pacific Biosciences SEQUEL II (HiFi) and Illumina NovaSeq 6000 (RNA-Seq) instruments. Hi-C data were also generated from thorax tissue of idTheAtra3 using the Arimav2 kit and sequenced on the Illumina NovaSeq 6000 instrument.

### Genome assembly, curation and evaluation

Assembly was carried out with Hifiasm ( Cheng *et al.*, 2021) and haplotypic duplication was identified and removed with purge_dups ( Guan *et al.*, 2020). One round of polishing was performed by aligning 10X Genomics read data to the assembly with Long Ranger ALIGN, calling variants with FreeBayes ( Garrison & Marth, 2012). The assembly was then scaffolded with Hi-C data ( Rao *et al.*, 2014) using YaHS ( Zhou *et al.*, 2023). The assembly was checked for contamination and corrected as described previously ( Howe *et al.*, 2021). Manual curation was performed using HiGlass ( Kerpedjiev *et al.*, 2018) and Pretext ( Harry, 2022). The mitochondrial genome was assembled using MitoHiFi ( Uliano-Silva *et al.*, 2023), which runs MitoFinder ( Allio *et al.*, 2020) or MITOS ( Bernt *et al.*, 2013) and uses these annotations to select the final mitochondrial contig and to ensure the general quality of the sequence.

A Hi-C map for the final assembly was produced using bwa-mem2 ( Vasimuddin *et al.*, 2019) in the Cooler file format ( Abdennur & Mirny, 2020). To assess the assembly metrics, the *k*-mer completeness and QV consensus quality values were calculated in Merqury ( Rhie *et al.*, 2020). This work was done using Nextflow ( Di Tommaso *et al.*, 2017) DSL2 pipelines “sanger-tol/readmapping” ( Surana *et al.*, 2023a) and “sanger-tol/genomenote” ( Surana *et al.*, 2023b). The genome was analysed within the BlobToolKit environment ( Challis *et al.*, 2020) and BUSCO scores ( Manni *et al.*, 2021; Simão *et al.*, 2015) were calculated.

Table 3 contains a list of relevant software tool versions and sources.

**Table 3.** Software tools: versions and sources.

Software tool	Version	Source
BlobToolKit	3.4.0	https://github.com/blobtoolkit/blobtoolkit
BUSCO	5.3.2	https://gitlab.com/ezlab/busco
Hifiasm	0.16.1-r375	https://github.com/chhylp123/hifiasm
HiGlass	1.11.6	https://github.com/higlass/higlass
Merqury	MerquryFK	https://github.com/thegenemyers/MERQURY.FK
MitoHiFi	2	https://github.com/marcelauliano/MitoHiFi
PretextView	0.2	https://github.com/wtsi-hpag/PretextView
purge_dups	1.2.3	https://github.com/dfguan/purge_dups
sanger-tol/genomenote	v1.0	https://github.com/sanger-tol/genomenote
sanger-tol/readmapping	1.1.0	https://github.com/sanger-tol/readmapping/tree/1.1.0
YaHS	yahs-1.1.91eebc2	https://github.com/c-zhou/yahs

### Genome annotation

The BRAKER2 pipeline ( Brůna *et al.*, 2021) was used in the default protein mode to generate annotation for the *Thecophora atra* assembly (GCA_937620795.1) in Ensembl Rapid Release.

### Wellcome Sanger Institute – Legal and Governance

The materials that have contributed to this genome note have been supplied by a Darwin Tree of Life Partner. The submission of materials by a Darwin Tree of Life Partner is subject to the **‘Darwin Tree of Life Project Sampling Code of Practice’**, which can be found in full on the Darwin Tree of Life website here. By agreeing with and signing up to the Sampling Code of Practice, the Darwin Tree of Life Partner agrees they will meet the legal and ethical requirements and standards set out within this document in respect of all samples acquired for, and supplied to, the Darwin Tree of Life Project. 

Further, the Wellcome Sanger Institute employs a process whereby due diligence is carried out proportionate to the nature of the materials themselves, and the circumstances under which they have been/are to be collected and provided for use. The purpose of this is to address and mitigate any potential legal and/or ethical implications of receipt and use of the materials as part of the research project, and to ensure that in doing so we align with best practice wherever possible. The overarching areas of consideration are:

• Ethical review of provenance and sourcing of the material

• Legality of collection, transfer and use (national and international) 

Each transfer of samples is further undertaken according to a Research Collaboration Agreement or Material Transfer Agreement entered into by the Darwin Tree of Life Partner, Genome Research Limited (operating as the Wellcome Sanger Institute), and in some circumstances other Darwin Tree of Life collaborators.

## Data Availability

European Nucleotide Archive: *Thecophora atra*. Accession number PRJEB51036; https://identifiers.org/ena.embl/PRJEB51036. ( Wellcome Sanger Institute, 2022) The genome sequence is released openly for reuse. The *Thecophora atra* genome sequencing initiative is part of the Darwin Tree of Life (DToL) project. All raw sequence data and the assembly have been deposited in INSDC databases. Raw data and assembly accession identifiers are reported in Table 1.

## Author information

Members of the Natural History Museum Genome Acquisition Lab are listed here: https://doi.org/10.5281/zenodo.4790042.

Members of the University of Oxford and Wytham Woods Genome Acquisition Lab are listed here: https://doi.org/10.5281/zenodo.4789928.

Members of the Darwin Tree of Life Barcoding collective are listed here: https://doi.org/10.5281/zenodo.4893703.

Members of the Wellcome Sanger Institute Tree of Life programme are listed here: https://doi.org/10.5281/zenodo.4783585.

Members of Wellcome Sanger Institute Scientific Operations: DNA Pipelines collective are listed here: https://doi.org/10.5281/zenodo.4790455.

Members of the Tree of Life Core Informatics collective are listed here: https://doi.org/10.5281/zenodo.5013541.

Members of the Darwin Tree of Life Consortium are listed here: https://doi.org/10.5281/zenodo.4783558.
