# Supplementary material for: The genome sequence of a conopid fly, Thecophora atra (Fabricius, 1775)
Source: Wellcome Open Res. 2023 Aug 21;8:358. [Version 1] doi: 10.12688/wellcomeopenres.19902.1 (PMC11320186; doi:10.12688/wellcomeopenres.19902.1)
Supplement: Peer review history

# Reviewer report: Reviewer response for version 1

**DOI:** 10.21956/wellcomeopenres.22037.r91098

**Published:** 2024-08-12

**Authors:** Jerome H L Hui

**Affiliations:** 1 The Chinese University of Hong Kong, Hong Kong, Hong Kong

In this data note, Mitchell and colleagues obtained the genomic resource of conopid fly, *Thecophora atra *(Fabricius, 1775). According to the NatureSpot and NBN Atlas, this species is widespread in Britain. There are only limited molecular data of this species prior to this report. Therefore, this new genome resource will be useful for further studies, ranging from species identification, revealing their population structure and biogeography, unravelling the effect of climate change on them, as well as understanding their evolution with other insects.

 This genome resource is excellent from the summary statistics, with high BUSCO scores, high sequence continuity (scaffold N50), and majority of sequences contained on the 3 pseudochromosomes (plus 2 sex chromosomes and mitochondrion). All in all, this is a valuable contribution.

Are sufficient details of methods and materials provided to allow replication by others?

Yes

Is the rationale for creating the dataset(s) clearly described?

Yes

Are the datasets clearly presented in a useable and accessible format?

Yes

Are the protocols appropriate and is the work technically sound?

Yes

Reviewer Expertise:

Genomics, evolution, invertebrates

I confirm that I have read this submission and believe that I have an appropriate level of expertise to confirm that it is of an acceptable scientific standard.

# Reviewer report: Reviewer response for version 1

**DOI:** 10.21956/wellcomeopenres.22037.r87475

**Published:** 2024-08-12

**Authors:** Ravikumar Dodiya

**Affiliations:** 1 Sardarkrushinagar Dantiwada Agricultural University, Sardarkrushinagar, Dantiwada, India

*Thecophora atra* (Fabricius, 1775) is a black fly from the family Conopidae, known as thick-headed flies. It is distinguished from *T. fulvipes* by its smaller size and unique leg colorations. The species is parasitic, laying eggs in halictid bees, with larvae feeding internally. *T. atra* inhabits chalky, calcareous grasslands and is distributed widely across Britain and Ireland, especially in southern regions.

 A high-quality genome of *T. atra *was sequenced using one male specimen from Hartslock Nature Reserve. The final assembly spans 354.2 Mb, organized into 22 scaffolds, with 99.94% of sequences mapped to chromosomes. The genome was annotated, identifying 30,620 protein-coding genes. This work, part of the Darwin Tree of Life Project, aids research on taxonomy, phylogeny, and biology of *T. atra* and related taxa.

Are sufficient details of methods and materials provided to allow replication by others?

Yes

Is the rationale for creating the dataset(s) clearly described?

Yes

Are the datasets clearly presented in a useable and accessible format?

Yes

Are the protocols appropriate and is the work technically sound?

Yes

Reviewer Expertise:

Biological control

I confirm that I have read this submission and believe that I have an appropriate level of expertise to confirm that it is of an acceptable scientific standard.

# Reviewer report: Reviewer response for version 1

**DOI:** 10.21956/wellcomeopenres.22037.r91101

**Published:** 2024-08-06

**Authors:** Darren J Obbard

**Affiliations:** 1 University of Edinburgh, Edinburgh EH, UK

This data note reports the sequencing and assembly of the genome of the conopid *Thecophora atra* as part of the “Darwin Tree of Life” programme. In common with other data notes from this research effort, the reporting is standardised and quite brief. As such, I have very few comments to make. 

 The approach is state-of-the-art, the raw data appear to be of a suitably high quality, and the assembly methods are appropriate. The public availability of raw data and genome assembly are appropriate. The resulting genome is likely to be of very high quality, and I have no doubt that it will be of great value to any researchers working on this fascinating group of flies, or on the comparative or evolutionary genomics of insects more generally. 

 The introduction is well written, and rich in interesting a relevant biological detail. I particularly like the information on identification and habitat, although I feel it would be additionally valuable to have more information on the geographic distribution and phenology elsewhere in the species range. Is it the same, or different to the British isles? I think there should also be a statement on the conservation status (if any) of the species. As always, I also think it would be nice to have a couple of good photos of male and female in life.

 Having read the first reviewer’s comments, I echo their call for greater clarity on the collections, sexes, and identification. I am also surprised by the very large number of genes (this seems unprecedented for a dipteran?), and would like to see some comment on what proportion of these are likely to be (e.g.) TEs?. Finally, it seems strange that the associated *Spiroplasma *endosymbiont assembly (idTheAtra2.Spiroplasma_sp_1) is not mentioned.

Are sufficient details of methods and materials provided to allow replication by others?

Partly

Is the rationale for creating the dataset(s) clearly described?

Yes

Are the datasets clearly presented in a useable and accessible format?

Yes

Are the protocols appropriate and is the work technically sound?

Yes

Reviewer Expertise:

Evolutionary genetics and genomics of insects and their pathogens

I confirm that I have read this submission and believe that I have an appropriate level of expertise to confirm that it is of an acceptable scientific standard, however I have significant reservations, as outlined above.

# Reviewer report: Reviewer response for version 1

**DOI:** 10.21956/wellcomeopenres.22037.r66176

**Published:** 2023-09-22

**Authors:** Rudolf Meier

**Affiliations:** 1 Center for Integrative Biodiversity Discovery, Leibniz Institute for Evolution and Biodiversity Science, Berlin, Germany

This is another valuable draft genome from the Darwin Tree of Life initiative. I have a few suggestions. Given that this a data note, some may not be directly relevant because they would lead to more in-depth analysis.

 (1) The abstract should be more informative. The genome is not really based only on one individual male, because it was assembled using a transcriptome from another specimen.

 (2) Ensembl may have identified 30,620 protein coding genes but Drosophila has ca. 19,000 and Culex 23,900. The unusually large number of genes reported here should be explained/discussed. Is this generally observed with the Ensembl  gene prediction pipeline? The mitochondrial genome is also longer than usual in Diptera.

 (3) “The specimen used for sequencing was confirmed as T. atra using DNA barcodes”. This requires more information. There are 10 barcodes filed under this species name in BOLDSYSTEM and they are assigned to two different BINs. It is thus very important that details on the barcode match is provided (e.g., matched to which sequence with which identity and overlap).

 (4) Overall, the information on how the specimens were identified and where the vouchers are deposited should be improved. Here is the current description:

 “Thecophora atra specimens (idTheAtra2 and idTheAtra3 NHMUK014444821) were collected from Hartslock Nature Reserve, Oxfordshire, UK (latitude 51.51, longitude –1.11) on 2020-08-20 using an aerial net. The specimens were collected and identified by Ryan Mitchell (Natural History Museum) and preserved on dry ice… The specimen used for RNA sequencing (specimen ID Ox000734, ToLID idTheAtra1) was collected from Wytham Woods, Oxfordshire (biological vice-county Berkshire), UK (51.766, –1.309) on 2020-08-03 by netting. This specimen was collected and identified by Steven Falk (independent researcher)."

 The questions are:

- There is only one collection number for both Hartslock specimens? Where are the vouchers. What is the sex of the second specimen from Hartslock.
- Is the identification based on morphology and DNA (and what are the details on the latter)
- Was the identification of the specimen from Wytham Woods also confirmed with a barcode and which one?  If not, the transcriptome COI should be compared to a barcode database.
- Presumably the RNA extraction was destructive or is there a voucher? Was it a male or female?

Variation in Diptera genome sizes is often due to transposable elements. Was there an analysis of TE proportion and composition? Similarly interesting would be looking for conserved broad-scale linkage maps that have been found when comparing drosophilid and tephritid genomes (https://academic.oup.com/aesa/article/94/6/936/8693).

 Lastly, the collecting details for the main specimen is repeated three times.

Are sufficient details of methods and materials provided to allow replication by others?

Partly

Is the rationale for creating the dataset(s) clearly described?

Yes

Are the datasets clearly presented in a useable and accessible format?

Yes

Are the protocols appropriate and is the work technically sound?

Yes

Reviewer Expertise:

Entomology

I confirm that I have read this submission and believe that I have an appropriate level of expertise to confirm that it is of an acceptable scientific standard, however I have significant reservations, as outlined above.
